# Molecular Phylogeography and Evolutionary History of the Endemic Species *Corydalis hendersonii* (Papaveraceae) on the Tibetan Plateau Inferred From Chloroplast DNA and ITS Sequence Variation

**DOI:** 10.3389/fpls.2020.00436

**Published:** 2020-04-08

**Authors:** Qien Li, Xiao Guo, Junfeng Niu, Dongzhu Duojie, Xianjia Li, Lars Opgenoorth, Jiabin Zou

**Affiliations:** ^1^ National Engineering Laboratory for Resource Developing of Endangered Chinese Crude Drugs in Northwest of China, Key Laboratory of the Ministry of Education for Medicinal Resources and Natural Pharmaceutical Chemistry, College of Life Sciences, Shaanxi Normal University, Xi'an, China; ^2^ Tibetan Medicine Research Center of Qinghai University, State Key Laboratory of Tibetan Medicine Research and Development, Qinghai University Tibetan Medical College, Xining, China; ^3^ State Key Laboratory of Tibetan Medicine Research and Development, Qinghai Tibetan Medicine Research Institute, Xining, China; ^4^ Department of Ecology, University of Marburg, Marburg, Germany; ^5^ Swiss Federal Research Institute WSL, Birmensdorf, Switzerland

**Keywords:** *Corydalis hendersonii* Hemsl., genetic variation, intraspecific diversification, phylogeography, quaternary climatic oscillations, refugium

## Abstract

In response to past climatic changes, the species with different habits or adaptive traits likely have experienced very different evolutionary histories, especially for species that restricted to high mountain areas. In order to trace how Quaternary climatic oscillations affected range distributions and intraspecific divergence of such alpine plants on the Tibetan Plateau, here, we investigated maternally inherited chloroplast DNA (cpDNA) markers and biparentally inherited nuclear ribosomal internal transcribed spacer (ITS) DNA variations and aimed to explore the phylogeographic history of the endemic alpine species *Corydalis hendersonii* Hemsl. (Papaveraceae). We sequenced four cpDNA fragments (*trn*S-*trn*G, *trn*T-*trn*L, *atp*H-*atp*I, and *psb*E*-pet*L) and also the nuclear (ITS) region in 368 individuals from 30 populations across the species' range. The network and phylogenetic analysis based on cpDNA variations identified 15 chlorotypes that cluster into three distinct clades. However, our nuclear DNA results demonstrated that there were four genetic/geographical groups within *C. hendersonii*. Some common and highly divergent cpDNA and ITS haplotypes were distributed in the populations of central and northeastern Tibetan Plateau, and the highest nucleotide diversity and genetic differentiation were detected in the central region. Demographic tests further indicated that the populations of southwestern and western Tibet may have experienced recent range expansion, which most likely occurred during the last glacial maximum (LGM) and continued its expansion after the beginning of the Holocene. These two different groups of this species may have derived from potential refugia that existed in the central and/or northeastern regions of Tibet during recent interglacial periods. In addition, our AMOVA analyses detected high genetic differentiation along with the whole sampling range. Also, distinct phylogeographic structures were detected among populations of *C*. *hendersonii* based on both of cpDNA and ITS variation. These findings shed new light on the importance of climatic oscillations during Quaternary and complex local topography as causes of intraspecific diversification and demographic changes within cold-tolerant herbs in the Tibetan Plateau biodiversity hotspot.

## Introduction

Climate oscillations during the Quaternary glaciations have modified the geographical distributions of most species in the temperate zone of the northern hemisphere markedly, generating varying degrees of range expansion and contraction. Such demographic changes undoubtedly shaped the geographical patterns of genetic variation within and among populations (Hewitt, 2000; Hewitt, 2004). In the past two decades, many phylogeographic studies have been conducted to explore this topic by tracing the spatial and genealogical distribution of genetic variation at the intra-speciﬁc level or among closely related species (Avise, 2000; Avise, 2008; Hickerson et al., 2010). The Tibetan Plateau is the highest and one of the most extensive plateaus in the world (Zheng, 1996; Zheng and Yao, 2004), which has become a “hotspot” of phylogeographical research (Li and Li, 1991; Sun et al., 2010; Qiu et al., 2011; Liu et al., 2014; Wen et al., 2014; Favre et al., 2015), owing to its complex local topography and geomorphology and past climatic ﬂuctuations. Moreover, the plateau, as well as the adjacent regions, are exceptional rich in endemic species and genera (Wu, 1988; Li and Li, 1993; López-Pujol et al., 2006), especially for alpine elements (Wu et al., 1995; Liu et al., 2000), comprising one of the world's “biodiversity hotspots” (Myers et al., 2000).

In response to past climatic changes, species with different life-history traits and adaptive traits likely have experienced very different evolutionary histories. For example, molecular-based research of numerous plant species shows that populations presently occurring on the plateau platform at high-altitude were derived from glacial refugia located at the edge of the north- and/or south-eastern plateau as well as the Hengduan Mountains. (e.g. Zhang et al., 2005; Meng et al., 2007; Chen et al., 2008a; Yang et al., 2008; Cun and Wang, 2010; Zou et al., 2012). Others, in turn, indicated *in situ* glacial survival on the southern plateau platform linked to the complex topography where populations could retreat to the valley bottom before expanding to the upper slopes during interglacials (Opgenoorth et al., 2009; Wu et al., 2010). In contrast, there is evidence that alpine plant species expanded their range during glacial periods while persisting at high-altitudes on the interior platform during warm periods (e.g. Wang et al., 2009a; Li et al., 2010; Shimono et al., 2010; Sun et al., 2010; Jia et al., 2011; Jia et al., 2012). In addition, deep intraspecific divergences and outstanding diversification were found in present-day plant populations, indicating that they have survived on the Tibetan Plateau throughout the glacial and interglacial periods of the Pleistocene (e.g. Wang et al., 2009a; Jia et al., 2011; Jia et al., 2012; Ren et al., 2017). Hybridization or introgression between different intraspecific lineages is common within these species (Qiu et al., 2011; Liu et al., 2012; Liu et al., 2014; Wen et al., 2014). Therefore, the intraspecific geographical pattern of genetic variation of the Tibetan Plateau could provide important insights into reconstructing their demographic history in more detail. However, how climate oscillations during the Quaternary affected range distributions and intraspecific divergence of alpine plants on the Tibetan Plateau remains largely unknown, especially for species that are widely distributed throughout the Tibetan Plateau but restricted to extremely high mountain areas. Because they should be the most sensitive to climate changes and would shed new light on phylogeographical history for alpine plants in this region.

*Corydalis hendersonii* Hemsl. (Papaveraceae), an alpine perennial herb endemic to the Tibetan Plateau, and is also a typical representative of the rare and endangered Tibetan medical plant. It currently grows at the river or rocky beach throughout the plateau, however, the distribution of this plant is limited to the high mountain areas at an altitude of 4,200–5,500 m (Wu et al., 1999; Zhang et al., 2008). Its ﬂowers are self-incompatible and predominantly pollinated by wild bees (Zhang et al., 2014). The mature seeds are suborbicular and very small (about 1.5–2 mm in diameter) (Wu et al., 1999), which presumably aid in their dispersal by wind. In addition, gravity and ants have been reported as the dispersal mechanisms of seed in its closely related species (Zhu et al., 2017). In the present study, we collected samples from 30 localities of *C. hendersonii* across its natural distribution areas within Tibet and examined the geographical patterns of chloroplast DNA (cpDNA) and nuclear ribosomal internal transcribed spacer (ITS) variations in the populations. Our major goals are: (1) to examine whether the interior Plateau serve as potential refugia for *C. hendersonii* during interglacial periods, (2) to reveal range dynamics of *C. hendersonii* in the history base on these two sets of genetic markers, and (3) to detect whether there is genetic divergence within the species largely due to the effects of climate oscillations during past glacial and interglacial periods.

## Materials and Methods

### Population Sampling

Leaf samples of *C. hendersonii* were collected from 368 individuals in 30 natural populations, covering the entire distribution range of this species. Five populations on the northeastern, eight populations on the central, thirteen populations on the southwestern, and five populations on the western Tibet were sampled (see **Table 1**). Two to 27 individuals were collected for each population, and all individuals were at least 50 m apart. Detailed information on the location, elevation, and sample size for each population is shown in **Table 1**.

**Table 1:** Sampling sites, sample size (*N*), chlorotypes distribution, estimates of haplotype diversity (*H*_E_), and nucleotide diversity (*π*) in 30 populations of *C. hendersonii*.

Population code	Sample location	Latitude	Longitude	Altitude	Chlorotypes	*H*_E_	*π* × 10^-3^	*N*
		(°N)	(°E)	(m)				
Northeastern populations								
1	Chamdo	31.56	97.67	4,704	C1(12), C2(2), C3(1)	0.362	0.15	15
2	Dengqen	31.21	95.60	4,830	C4(10)	0.000	0.00	10
3	Sog	31.88	94.49	4,925	C3(2)	0.000	0.00	2
4	Biru	31.48	93.68	4,800	C3(17), C5(1)	0.111	0.03	18
5	Amdo	32.87	91.93	5,623	C9(11)	0.000	0.00	11
Average							0.036	
Central populations								
6	Lhari	30.75	93.09	5,335	C3(10), C6(1), C7(1)	0.318	0.24	12
7	Sangri	29.66	92.45	4,864	C6(11)	0.000	0.00	11
8	Maldrogongkar	29.83	92.35	4,927	C3(2), C6(9), C8(1)	0.439	0.22	12
9	Maldrogongkar	30.42	92.21	4,969	C7(12)	0.000	0.00	12
10	Nagchu	30.61	91.93	4,850	C7(13)	0.000	0.00	13
11	Damxung	30.69	91.11	5,050	C7(11)	0.000	0.00	11
12	Lhunzhub	30.12	91.27	4,785	C3(17)	0.000	0.00	17
13	Lhasa	29.75	91.21	4,912	C11(15)	0.000	0.00	15
Average							0.058	
Southwestern populations								
14	Zhanang	28.97	91.32	5,135	C3(16)	0.000	0.00	16
15	Comai	28.80	91.77	4,961	C3(13), C10(1)	0.143	0.04	14
16	Comai	28.71	91.80	5,164	C3(4), C6(5)	0.556	0.34	9
17	Comai	28.35	91.77	5,213	C3(16)	0.000	0.00	16
18	Gonggar	28.99	91.09	5,128	C3(8)	0.000	0.00	8
19	Rinbung	29.17	90.23	5,266	C3(8)	0.000	0.00	8
20	Gyangze	28.89	90.19	5,224	C3(8)	0.000	0.00	8
21	Nagarze	28.63	91.19	5,422	C3(8)	0.000	0.00	8
22	Namling	29.63	89.47	5,326	C3(10)	0.000	0.00	10
23	Shigatse	29.46	89.04	4,884	C3(12)	0.000	0.00	12
24	Xaitongmoin	29.46	88.40	5,706	C3(10)	0.000	0.00	10
25	Ngamring	29.05	86.59	5,774	C3(11)	0.000	0.00	11
26	Nyima	32.75	87.65	5,396	C3(22), C12(4), C13(1)	0.325	0.10	27
Average							0.037	
Western populations								
27	Gerze	31.77	84.94	5,580	C3(10)	0.000	0.00	10
28	Gegyai	32.38	82.32	5,947	C3(11)	0.000	0.00	11
29	Burang	31.03	81.29	5,543	C3(12), C14(3)	0.343	0.10	15
30	Gar	31.66	80.12	5,460	C15(16)	0.000	0.00	16
Average							0.025	
Total						0.596	0.38	368

### DNA Extraction, Amplification, and Sequencing

Genomic DNA was isolated from approximately 20 mg of silica gel-dried leaf materials using either a QIAGEN DNeasy Plant Mini Kit (QIAGEN, Inc.,Valencia, CA, USA) or the modified CTAB procedure (Doyle and Doyle, 1990). Four cpDNA fragments (*trn*S-*trn*G, *trn*T-*trn*L, *atp*H-*atp*I, and *psb*E*-pet*L) and one nuclear ribosomal ITS fragments were amplified and sequenced following the suggested primers (Taberlet, 1991; Hamilton, 1999; Grivet et al., 2001; Gaskin and Wilson, 2007; Dong et al., 2012). Detailed information on the primer sequences is shown in the **Table S1**. Polymerase chain reaction (PCR) was performed with 25-μl volume and each reaction contained 15–20 ng DNA, 50 mM Tris-HCl, 1.5 mM MgCl_2_, 0.5 mM dNTPs, 2.5 μM of each primer, and 0.75 unit of Taq polymerase. The amplification and sequencing conditions for each primer pair are shown in **Table S2**. Singletons were verified by repeated amplification and re-sequencing from the same DNA. Sequences were aligned by Clustal X (Thompson et al., 1997) or Clustal W implemented in MEGA 6.0 (Tamura et al., 2013), and all gaps (indels) were coded as binary states (0 or 1) using the GAPCODER software (Young and Healy, 2003). All cpDNA sequences were identified to different haplotypes using DnaSp version 5.0 (Librado and Rozas, 2009). For ITS gene analysis, heterozygous positions were phased by the software package Phase 2.1 (Stephens et al., 2001; Stephens and Donnelly, 2003) to deduce haplotypes, allowing for recombination among haplotypes and using a posterior probability cutoff of 0.95. All sequences have been deposited in the GenBank under accession numbers MT023736–MT023784.

### Phylogeographic Analyses

In order to search for partitions of sampling sites that were genetically homogenous but maximally differentiated from each other, firstly, a spatial analysis of molecular variance (SAMOVA) was conducted with SAMOVA 2.0 (Dupanloup et al., 2002), based on both cpDNA and ITS dataset respectively. Based on value *K* that needs to be optimized, this method uses simulated annealing procedures to seek the best clustering option that can be defined between groups of populations among group genetic variation coefficients (*F*_CT_). We tested values for *K* in the range of 2–16, and the initial condition was set to 100 with 10,000 iterations. The conﬁguration with the largest associated *F*_CT_ value after the 100 independent simulated annealing processes was retained as the best grouping of populations.

For each population and the species overall, haplotype (*H*_E_) and nucleotide diversity (*π*) were estimated by the software DnaSp version 5.0 (Librado and Rozas, 2009) for both of cpDNA and ITS dataset. The average gene diversity within populations (*H*_S_), total gene diversity (*H*_T_), and the coefficients of differentiation between *G*_ST_ and *N*_ST_ were estimated for four genetic/geographical groups (as identiﬁed by ITS SAMOVA analysis in this study) as well as the species overall based on cpDNA and ITS markers respectively, using the PERMUT software with 1,000 permutations (Pons and Petit, 1996). As PERMUT software needed at least three individuals per population, one population (population 3) was excluded during the analysis due to its limited individuals (only two individuals). We compared *G*_ST_ and *N*_ST_ using the U-statistic, which was approximated by a Gaussian variable by taking into account the covariance between *G*_ST_ and *N*_ST_, and a one-sided test (Pons and Petit, 1996). The former consider only haplotype frequencies while *N*_ST_ also takes into account differences between haplotypes. When *N*_ST_ is larger than *G*_ST_, the phylogeographic structure is obvious, which indicates that closely related haplotypes are found more often in the same area than less closely related haplotypes (Pons and Petit, 1996). Hierarchical partitioning of diversity between populations was also estimated based on analysis of molecular variance (AMOVA) (Excoffier et al., 1992) using the program ARLEQUIN version 3.0 (Excoffier et al., 2005), with significance tests based on 10,000 permutations. *F*_ST_, generally expressed as the proportion of genetic diversity due to allele frequency differences among populations, was used to assess population differentiation within and between four genetic groups. *F*_ST_ values were estimated by the analysis of AMOVA using ARLEQUIN version 3.1.1 (Excoffier et al., 2005), with significance tests based on 10,000 permutations.

A Bayesian method was further used to assess the population genetic structure of *C. hendersonii* based on ITS sequences data, as implemented in the software STRUCTURE version 2.3.4 (Hubisz et al., 2009). STRUCTURE also gives the probabilities of assignment of each individual to each cluster. We used these probabilities to infer the membership of each individual in its most probable cluster. Before we performed structure analysis, sites that showed significant linkage after Bonferroni correction were removed (Heuertz et al., 2006). To infer the number of cluster, a fully Bayesian process described by Pritchard et al. (2000) was run with different values for the number of clusters (1 ≤ *K* ≤ 8). We performed 20 runs with a burn-in of 200,000 and 500,000 iterations, and used the program DISTRUCT version 1.1 (Rosenberg, 2004) to generate graphical representations of the data. The most likely number of clusters was estimated with the original method of Pritchard et al. (2000) and with the △*K* statistics given in Evanno et al. (2005).

### Phylogenetic Analyses

Genealogical relationships among cpDNA and ITS haplotypes were constructed respectively using median-joining networks with NETWORK version 4.6.0.0 (Bandelt et al., 1999). In order to confirm the haplotype relationships suggested by NETWORK, we also performed a maximum likelihood (ML) analysis under the GTRGAMMA substitution model, using the software RAXML version 7.2.6 (Stamatakis, 2006), respectively with two species of *Corydalis* (*C*. *dasyptera* and *C*. *decumbens*) as outgroups. The rapid-bootstrapping algorithm (1,000 bootstrap replicates) with the thorough ML search option (200 independent searches, starting from every fifth bootstrap replicate) was used to search for the best-scoring ML tree. A Bayesian approach was further used to estimate the relationship between haplotypes as implemented in the software MrBayes version 3.2.7 (Ronquist and Huelsenbeck, 2003; Ronquist et al., 2012). Two independent runs with four chains each (one cold, three heated) were run using the General Time Reversible (GTR) model and gamma-distributed rate variation across sites with a proportion of invariant sites. Markov chain Monte Carlo (MCMC) searches were run for 10,000,000 generations and trees were sampled every 100 generations. Analyses were continued until the average standard deviation of split frequencies (ASDoSF) between the two runs was <0.01. After the first 25,000 trees were discarded as burn-in based on the stationarity of likelihood values, a majority-consensus tree, as well as posterior probabilities, were obtained from the estimated distribution of trees. The software packages TreeView and MEGA 6.0 (Tamura et al., 2013) were used for visualizing the phylogenetic tree.

### Demographic Analyses

Several methods were used to detect demographic history based on plastid DNA data sets. Firstly, tests of selective neutrality were used to infer potential population growth and expansion, including Tajima's *D* (Tajima, 1989) and Fu's *F*_S_ (Fu and Li, 1993). For each genetic group as well as the species overall, we also tested the null hypothesis of spatial expansion using mismatch distribution analysis (MDA) in ARLEQUIN version 3.0 (Excoffier et al., 2005). The goodness-of-ﬁt was tested with the sum of squared deviations (SSD) between observed and expected mismatch distributions, and Harpending's (1994) raggedness index (*H*_Rag_), using 1,000 parametric bootstrap replicates. When the hypothesis of rapid expansion was not rejected (see *Results*), we converted the parameter value *τ* into estimates of time since expansion (*t*) in years according to the formula *t* = *τ*/2*u* (Rogers and Harpending, 1992). In this formula, the parameter *u* is the mutation rate which can be calculated as *u* = 2*μkg*, where *μ* is the substitution rate per nucleotide site per year, *k* is the average sequence length, and *g* is the generation time in years. Maximal and minimum substitution rates, which were chosen based on the lower and upper ranges for previously estimated (synonymous) substitution rates of 1.0 × 10^-9^ and 8.24 × 10^-9^ substitutions per site per year for cpDNA in angiosperms (Wolfe et al., 1987; Richardson et al., 2001), were used to estimate the expansion times respectively. Based on our observations on the age of ﬁrst reproduction of *C. hendersonii* in cultivation at Qinghai University, we took the generation time to be 5 years.

In addition, we analyzed our cpDNA data with the extended Bayesian skyline plot (EBSP) method, a Bayesian approach for inferring past population size fluctuations from genetic data, as implemented in the software BEAST version 2.3.2 (Bouckaert et al., 2014). Building on the previous Bayesian skyline plot (BSP) approach, EBSP uses a piecewise-linear model and Markov chain Monte Carlo (MCMC) methods to reconstruct a populations' demographic history (Heled and Drummond, 2008). Alignments for each of the four genetic groups as well as all populations were loaded separately into the Bayesian Evolutionary Analysis Utility tool (BEAUti v. 2.3.2) in NEXUS format. A “Gamma Category Count” of four and strict molecular clock were selected, and the “Coalescent Extended Bayesian Skyline” process was used for all analyses. All other operator settings were set as default. Posterior distributions of parameters were approximated using Monte Carlo Markov chains (MCMC) of 100 million generations each, with parameters sampled every 10,000 generations and the first 10 million samples were discarded as burn-in. Log ﬁles were analyzed in Tracer v. 1.4 (Rambaut and Drummond, 2007) to assess convergence and to conﬁrm that the effective sample sizes for all parameters were larger than 200, indicating that stationary distributions had been reached. Demographic reconstructions were then plotted in R software version 3.2.3.

### Species Distribution Modeling

In order to examine the role that past climatic oscillation might play in controlling biogeographic patterns within *C. hendersonii*, we employed ecology niche models to predict the distributions of *C. hendersonii* at the present time, the Last Glacial Maximum (LGM; ~ 21 000 years before present) and the Last Inter-Glacial (LIG; ~ 120 000–140 000 years before present), respectively. In addition to the distribution records in this study (see **Table 1**), GPS data from 60 localities of museum records (see **Table S3**) were used to generate species distribution models for *C. hendersonii*. Ecological niche models with inappropriately complex variables might be oversized, overfitted, or redundant (Parolo et al., 2008; Swanepoel et al., 2013). To increase abilities in building high accuracy predictions and in identifying the critical predictors constraining the species' distribution, we implemented an optimized selection of 19 bioclimatic variables (**Table S4**) from WorldClim database (Hijmans et al., 2005), based on sample-size-corrected Akaike information criteria (AICc) (Warren and Seifert, 2011; Warren et al., 2014). The optimized variable selection was processed in R software with package ‘‘MaxentVariableSelection'' (Jueterbock, 2015; Jueterbock et al., 2016), by excluding variables with a relative contribution score <5% or a correlation of >0.9 with other variables (**Table S4**). The model with the lowest AICc was considered to have the most appropriate complexity (Warren and Seifert, 2011; Jueterbock et al., 2016), thus the variables included in this model were selected to build the final model for *C. hendersonii* habitat (**Table S4**). Species potential distributions were generated under the maximum entropy model implemented in the program MaxEnt 3.3.3k (Phillips et al., 2006; Phillips and Dudik, 2008). We generated 10 jackknife replicates with deletion of 20% of species occurrence localities for each analysis to account for error in the predictive modeling. Replicate was run for 500 interactions with a convergence threshold of 10^-5^. We conﬁgured the machine-learning algorithm to use 75% of species records for training and 25% for testing the model. The software DIVA-GIS v7.5 (Hijmans et al., 2005) was used to create the graphics of species distributional ranges based on raster cells from original species distribution modeling, with a logistic habitat suitability index ranging from the lowest “0” to the highest “1.”

## Results

### Population Grouping

**Figure 1:**
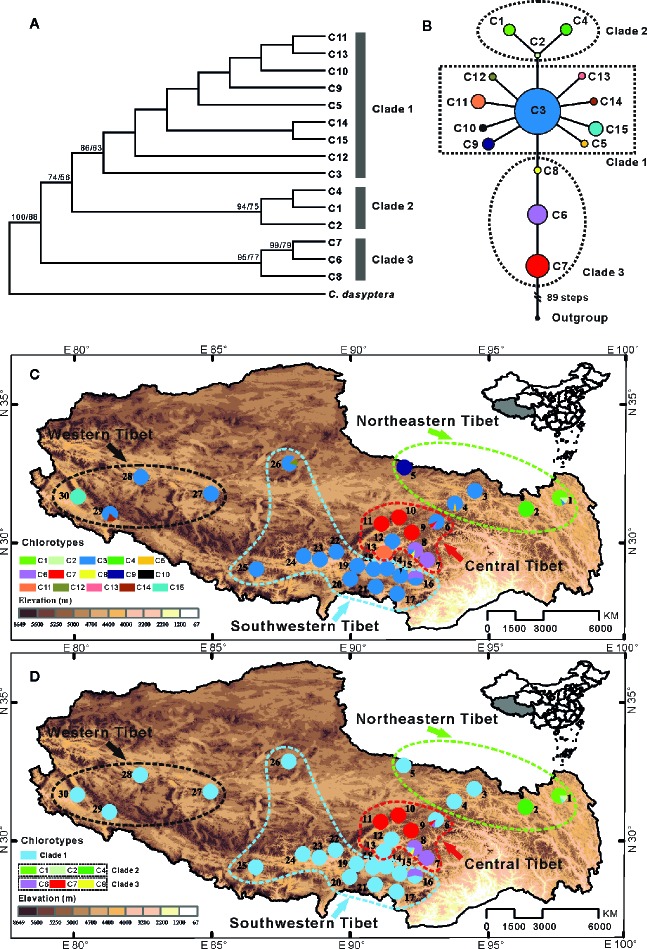
**(A)** Bayesian consensus tree based on 15 chlorotypes (C1–C15) identified in *C. hendersonii*. Numbers next to nodes indicated posterior probabilities and bootstrap values (only the values >50% are shown) based on Bayesian and maximum-likelihood (ML) analysis respectively. **(B)** Network of 15 chlorotypes for *C. hendersonii*. Each circle means a single haplotype sized in proportion to its frequency. Different colors denote different haplotypes. **(C)** Geographic distribution of all 15 chlorotypes detected in *C. hendersonii* (see **Table 1** for population codes); **(D)** Geographic distribution of six haplotypes of clade 2 and clade 3 relative to all of clade 1.

The SAMOVA analysis revealed that the cpDNA dataset of *C. hendersonii* could be partitioned into four genetic/geographical groups. *F*_CT_ values increase progressively with *K*. When *K* was greater or equal to 5, at least one member of the group contained a single population of *C. hendersonii* (**Table S5**), indicating that the group structure was disappearing. When *K* = 4, most of the populations formed a group, six populations located in central Tibet were separated into two groups (9, 10 and 11, 7, 8 and 16), only two populations (populations 1 and 2) from the northeastern Tibet formed the last one group. However, this population grouping showed a weak geographical relationship between four genetic groups. In the SAMOVA analysis based on ITS dataset, *F*_CT_ values also increased progressively as *K* was increased. For the first three *F*_CT_ values, the *F*_CT_ value was highest when *K* = 4. When *K* was between 5 and 16, at least one group consisted of only a single population. Therefore, in our dataset, *C. hendersonii* population could optimally be placed into four groups. These four groups included the northeastern group (populations 1 to 5), central group (populations 6 to 13), southwestern group (populations 14 to 26), and western group (populations 27 to 30) (**Figure 1** and **Table S6**). This grouping scenario detected better possible breaks among populations, suggesting a relatively strong geographical relationship between four genetic groups. Therefore, this population grouping was selected and used in our following data analysis.

### CpDNA Diversity, Haplotype Distribution, and Phylogeographic Structure

The four combined cpDNA fragments *trn*S-*trn*G, *trn*T-*trn*L, *atp*H-*atp*I, and *psb*E*-pet*L had a total length of 3,268 bp and yielded five indels (1–5 bp in length) and eight substitutions. The sequence length and variation for each chloroplast DNA fragment are shown in the **Table S7**. A total of 15 chlorotypes (C1–C15) were identified among all sampled 368 individuals of *C. hendersonii* across the 30 surveyed populations (**Table 1** and **Table S8**). Consequently, the cpDNA data revealed relatively low estimates of haplotype diversity (*H*_E_ = 0.596 × 10^-3^) and nucleotide diversity (*π =* 0.38 × 10^-3^) at the species level. In each genetic group, the average diversity was highest in the central populations (*π* = 0.058 × 10^-3^) and lowest in western populations (*π* = 0.025 × 10^-3^), while populations from the eastern (*π* = 0.036 × 10^-3^) and southwestern (*π* = 0.037 × 10^-3^) Tibet showed intermediate levels (**Table 1**).

Three distinct clades were discerned in the 15 chlorotypes by the phylogenetic analysis (**Figure 1A**) and parsimony network (**Figure 1B**). The first clade included nine chlorotypes (C3, C5, and C9–15), and the network analysis showed that the other eight chlorotypes have a star-like distribution around chlorotypes C3, which was widespread in 22 of the 30 surveyed populations. Especially the populations in southwestern and western Tibet were fixed only for this chlorotype (populations 12, 14, and 17–28). Three chlorotypes (C9, C11, and C15) in this clade were found in three isolated populations (populations 5, 13, and 30) with each of these populations fixed for only one chlorotype. Clade 2 contained three chlorotypes (C1, C2, and C4), which occurred in only two populations (populations 1 and 2) in the eastern region of Tibet. All three chlorotypes (C6, C7, and C8) that belonged to clade 3 were only found in the populations of the central plateau (populations 6 to 11, 16) at high frequency except chlorotypes C8, which was only recovered in one population (population 8) with low frequency (**Figures 1C, D**).

Among the four genetic groups of *C. hendersonii*, the total diversity (*H*_T_) and the average gene diversity (*H*_S_) were the highest for the central populations (*H*_T_ = 0.893, *H*_S_ = 0.095), which also showed the highest between-population differentiation (*G*_ST_ = 0.894) (**Table 2**). Signiﬁcant phylogeographic structures existed at the range-wide scale of *C. hendersonii* (*N*_ST_ = 0.894, *G*_ST_ = 0.854, P < 0.05), however, no obvious structure was detected within the four genetic groups because all comparisons failed to detect signiﬁcant larger *N*_ST_ values than *G*_ST_ (see **Table 2**). AMOVA analyses revealed that 87.70% of the species' total variation in cpDNA was distributed among populations (*F*_ST_ = 0.877, P < 0.0001); 76.76% of the variation was explained if the *C. hendersonii* populations were grouped into four ITS SAMOVA groups (**Table 3**). *F*_ST_ values within and between the four genetic groups estimated by AMOVA are given in **Table 4**. Populations from central Tibet appeared to be the most strongly differentiated lineage, with *F*_ST_ values as high as 0.2969, 0.4654, and 0.3985 with respect to northeastern, southwestern, and western populations. Populations from western Tibet showed relatively lower genetic differentiations compared to the populations from northeastern (*F*_ST_ = 0.2222, P < 0.0001) and southwestern (*F*_ST_ = 0.2305, P < 0.0001) Tibet. *F*_ST_ values among populations within each geographic region were lower than that between the four geographic regions, with the lowest value having been detected within the southwestern populations (*F*_ST_ = 0.0598, P < 0.0001).

**Table 2:** Estimates of average gene diversity within populations (*H*_s_), total gene diversity (*H*_T_), between-population differentiation (*G*_ST_), and number of substitution types (*N*_ST_) overall populations and within four different population groups.

Population groups	cpDNA					ITS			
	*H*_S_	*H*_T_	*G*_ST_	*N*_ST_		*H*_S_	*H*_T_	*G*_ST_	*N*_ST_
Northeastern populations (1–5)	0.095	0.811	0.883	0.932		0.525	0.726	0.277	0.242
Central populations (6–13)	0.095	0.893	0.894	0.899		0.829*	0.959*	0.136*	0.212 (NC)
Southwestern populations (14–26)	0.079	0.123	0.390 (NC)	0.419 (NC)		0.725*	0.802	0.096*	0.109
Western populations (27–30)	0.086	0.567	0.849	0.857		0.793*	0.895	0.114*	0.324
All populations	0.087	0.594	0.854	0.894*		0.729*	0.933*	0.219*	0.359*

Population numbers are shown in the parentheses. *P < 0.05; NC, not calculated.

**Table 3:** Analyses of molecular variance (AMOVA) based on cpDNA and ITS dataset from *C. hendersonii*.

Grouping	Source of variation	cpDNA		ITS	
		PV (%)	Fixation index	PV (%)	Fixation index
Total populations	Among populations	87.70	*F*_ST_ = 0.877*	28.30	*F*_ST_ = 0.283*
	Within populations	12.30		71.70	
SAMOVA groups	Among groups	76.76	*F*_ST_ = 0.920*	36.93	*F*_ST_ = 0.425*
	Among populations within groups	15.23	*F*_SC_ = 0.759*	5.60	*F*_SC_ = 0.156*
	Within populations	8.01	*F*_CT_ = 0.768*	57.47	*F*_CT_ = 0.369*

PV, percentage of variation; * represent P < 0.0001, 1000 permutations; F_CT_, correlation of haplotypes within groups relative to total; F_SC_, correlation within populations relative to groups; F_ST_, correlation within populations relative to total.

**Table 4:** Pairwise comparisons of *F*_ST_ within and between four different population regions of *C. hendersonii* based on cpDNA and ITS dataset.

Regions	cpDNA					ITS			
	Northeast	Center	Southwest	West		Northeast	Center	Southwest	West
Northeast	0.1487*					0.1039*			
Center	0.2969*	0.1890*				0.4655*	0.1813*		
Southwest	0.3129*	0.4654*	0.0598*			0.4652*	0.3029*	0.1996*	
West	0.2222*	0.3985*	0.2305*	0.0990*		0.2766*	0.3518*	0.2848*	0.2199*

*Represent P < 0.0001.

### Nuclear Gene Diversity, Haplotype Distribution, and Population Structure

By examining sequence variation of one ITS fragment, about 96% of the sampled 166 individuals across the 30 surveyed populations turned out to be heterozygotes. The 166 sequences of this nuclear gene region were 582 bp in aligned length and characterized by 25 substitutions, resulting in forty-nine haplotypes (H1–H49) (**Table S9**). The estimated nucleotide diversity (*π*) overall populations ranged from 0.57 × 10^-3^ to 7.84 × 10^-3^. In each genetic group, the average diversity was highest in the central populations (*π* = 4.56 × 10^-3^) and lowest in eastern populations (*π* = 1.70 × 10^-3^), and populations from the southwestern (*π* = 3.43 × 10^-3^) and western (*π* = 3.24 × 10^-3^) Tibet showed intermediate levels (**Table S10**).

**Figure 2:**
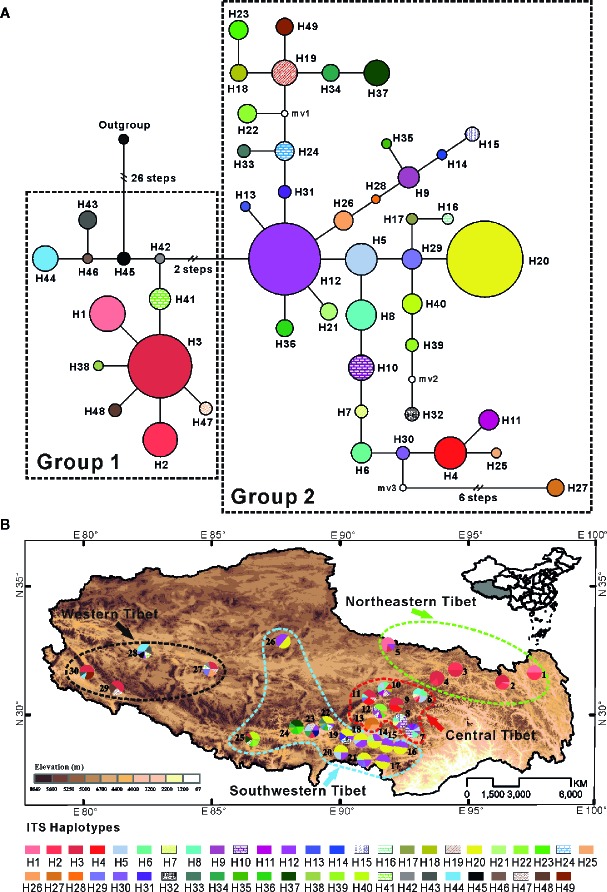
**(A)** Network of 49 ITS haplotypes for *C. hendersonii*. Each circle means a single haplotype sized in proportion to its frequency. Different colors denote different haplotypes. **(B)** Geographic distribution of all 49 ITS haplotypes detected in *C. hendersonii* (see **Table 1** for population codes).

Two distinct groups were discerned in the 49 ITS haplotypes by the parsimony network (**Figure 2A**). The first group included 12 haplotypes (H1–H3, H38, and H41–H48), and the network analysis showed that six (H1–H2, H38, H41, and H47–H48) of all 12 haplotypes have a star-like distribution around haplotype H3, which was widespread but mainly found in northeastern populations and western population. Haplotypes H1 and H2 only occurred in five northeastern populations (populations 1 to 5), another eight haplotypes (H41–H48) in this group were only found in four western populations (populations 27 to 30) (see **Figure 2** and **Table S10**). Group 2 contained all other 37 haplotypes, which were mainly found in central and southwestern populations. Especially the populations in central Tibet (populations 6–13) fixed most of them (H4–H29). Haplotypes H12 and H20, the former one seemed like an ancient haplotype due to its central place of the network, were widespread in the populations of the southwestern plateau at high frequency (**Figure 2** and **Table S10**), suggesting that there might be population expansion in this region during the past time. In the Bayesian and ML tree, although the posterior probabilities and bootstrap values were low and the ITS haplotypes belonged to the second group were failed to cluster together, all 12 haplotypes from the first group were cluster together with relatively high support (**Figure S1**).

Among the four genetic groups of *C. hendersonii*, the total diversity (*H*_T_) and the average gene diversity (*H*_S_) were the highest for the central populations (*H*_T_ = 0.959, *H*_S_ = 0.829). Signiﬁcant phylogeographic structures existed at the range-wide scale (*N*_ST_ = 0.359, *G*_ST_ = 0.219, P < 0.05) as well as central (*N*_ST_ = 0.212, *G*_ST_ = 0.136, P < 0.05) and western populations (*N*_ST_ = 0.324, *G*_ST_ = 0.114, P < 0.05), however, no obvious structure was detected within the northeastern and southwestern populations because all comparisons failed to detect signiﬁcant larger *N*_ST_ values than *G*_ST_ (**Table 2**). In contrast to the cpDNA results, AMOVA analyses revealed that the molecular difference among populations in the ITS data was low (28.30%) and accounts for 71.70% of the genetic variation within populations (**Table 3**). When the populations were partitioned into the four ITS SAMOVA groups, a relatively low level of the variation (57.47%) was observed within populations, but the variation among groups was slightly higher (36.93%) (**Table 3**). Thus, four genetic groups might be optimal for these populations. Analysis of population differentiation estimated by AMOVA showed that *F*_ST_ values between each pair of the four geographical regions were higher than that within each region (**Table 4**), however, the lowest differentiation (*F*_ST_ = 0.2766, P < 0.0001) was detected between northeastern and western groups.

**Figure 3:**
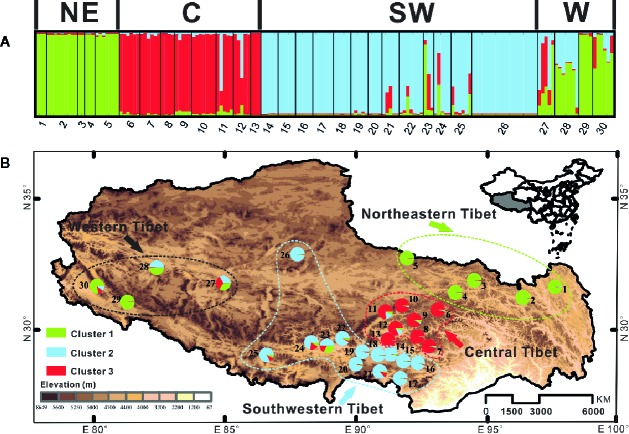
Bayesian clustering results of the structure analysis for ITS sequence data of 368 individuals (30 populations) of *C. hendersonii* form the Tibetan Plateau. **(A)** Histogram of the structure analysis for the model with *K* = 3; **(B)** Geographic origin of the 30 C*. hendersonii* populations and their color-coded grouping according to the structure analysis for the model with *K* = 3. NE, northeastern Tibet; C, central Tibet; SW, southwestern Tibet; W, western Tibet.

Genetic structure among populations of *C. hendersonii* was identified by the Bayesian clustering algorithm tests using the ITS dataset, which revealed that the most likely number of clusters was *K* = 3 when we used △*K* statistics. These results suggested a hierarchical structure in the data (see **Figure 3**). For *K* = 3, the first cluster mainly comprised individuals from northeastern and western populations, the other two clusters were each dominated by individuals from central and southwestern populations respectively (**Figure 3A**). However, few populations from southwestern region (populations 21 to 25) and the most of individuals from the western populations contained some contributions derived from other clusters, especially for that dominated by individuals from northeastern and central populations, showing signs of genetic admixture and suggesting that there might be high gene flow or the retention of ancestral polymorphism among those genetic groups. Their geographic distribution was relatively congruent with that of cpDNA in representing northeastern Tibet (populations 1 to 5) and central Tibet (populations 6 to 13), whereby samples from the cpDNA subclade 1 (see **Figure 1**) were further subdivided into “gene pools” from southwestern Tibet (populations 14 to 26) and western Tibet (populations 27 to 30) (**Figure 3B**).

### Demography Histories

**Figure 4:**
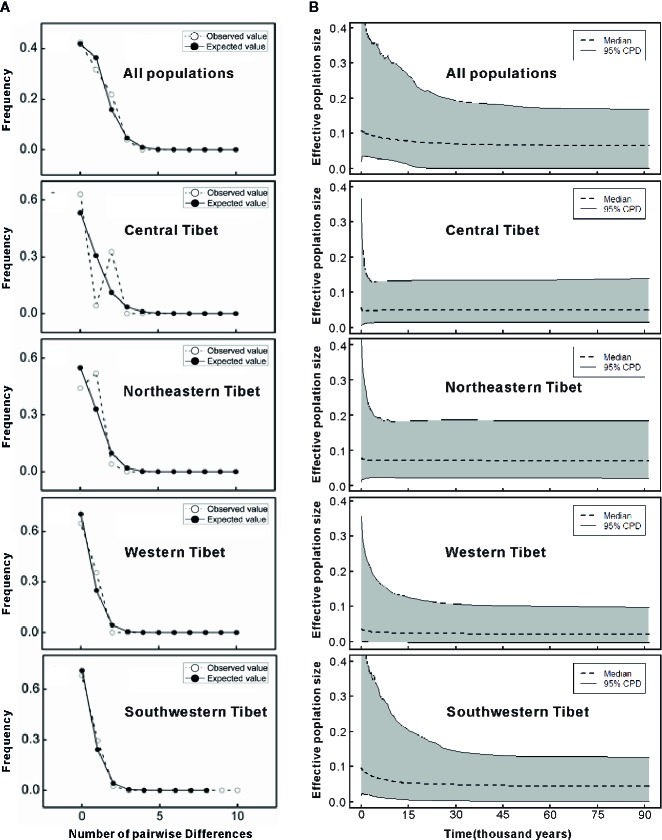
**(A)** Distribution of the number of pairwise nucleotide differences for cpDNA sequence data in all populations and in each genetic group of *C. hendersonii*. The solid line shows observed distributions of differences among chlorotypes, whereas the dashed line represents simulated distributions under a model of sudden (stepwise) demographic population expansion. **(B)** Extended Bayesian skyline plots (EBSP) for effective populations sizes (assuming generation time of 5 years) in all populations and in each genetic group of *C. hendersonii*. Dotted lines are the median values and the gray areas represent the boundary of the 95% central posterior density (CPD) intervals.

The mismatch distributions for chlorotypes of *C. hendersonii* in most cases were unimodal, except that of the northeastern and central populations, which were bimodal (**Figure 4A**). However, none of the observed distributions of haplotypes for each region reject the spatial expansion model (all *P* values > 0.05 based on SSD and *H*_Rag_; **Table 5**). Nevertheless, neutral tests detected slightly significant negative values of Tajima's *D* and Fu's *F*_S_ for southwestern populations (*D* = −0.1273, P < 0.05; *F*_S_ = −3.166, P < 0.1; **Table 5**) and western populations (*D* = −1.281, P < 0.1; *F*_S_ = −0.585, P < 0.1; **Table 5**) of *C. hendersonii*, and the newly derived haplotypes (clade 1) were mainly found in these populations (**Figures 1B, D**), indicating that the expansion model might be applicable. It should be noted that, because most of these demographic indices were non-signiﬁcant or lacking strong support, these presumptions should be treated with caution. Based on the highest and lowest mutation rates so far recorded for cpDNA in angiosperm species, we dated the spatial expansion of southwestern populations to glacial cycles likely occurred between 14 and 122 Kya (**Table 5**), whilst the demographic expansion of western populations might have happened more recently (between 3 and 27 Kya, **Table 5**).

**Table 5:** Estimates of neutrality tests and mismatch distribution analysis for pooled populations of *C. hendersonii* based on cpDNA sequence data.

Regions	Parameter (*τ*)	*t*_min_ (kya)	*t*_max_ (kya)	*SSD*	*H*_Rag_	Fu's *Fs*	Tajima's *D*
Northeast	0.014 (0.000–5.549)	NC	NC	0.041	0.079	2.759	1.573
Center	3.191 (0.000–46.691)	NC	NC	0.018	0.069	2.416	1.973
Southwest	3.000 (0.000–3.500)	14.890 (0.000–217.871)	122.693 (0.000–1,795.255)	0.005	0.653	−3.166**	−1.273*
West	0.703 (0.004–1.232)	3.280 (0.019–5.749)	27.030 (0.154–47.370)	0.023	0.192	−0.585*	−1.281*
Overall	0.000 (0.000–0.438)	NC	NC	0.443	0.065	−3.906	−0.602

SSD, sum of squared deviations; H_Rag_, Harpending's raggedness index; τ, time in number of generations elapsed since the sudden expansion episode; t, expansion time; Upper and lower 95% conﬁdence limits around estimates of τ and associated ranges of t are in parentheses; NC, not calculated. **P < 0.05; *P < 0.1.

However, further EBSP analysis showed similar demographical histories of *C. hendersonii* (**Figure 4B**). All populations revealed profiles with a stable size up to approximately 30–45 Kya flowed by a slowly increase towards the present (**Figure 4B**). Northeastern and central populations share a relatively small, stable ancestral size that showed little change during the past glacial cycles. Both southwestern and western populations showed the signal of expansion. However, the signal of expansion in southwestern populations was stronger and started earlier (around 45 to 30 Kya), western populations showed a weaker signal of expansion and started later (around 15 Kya) (**Figure 4B**).

### Species Distribution Change

**Figure 5:**
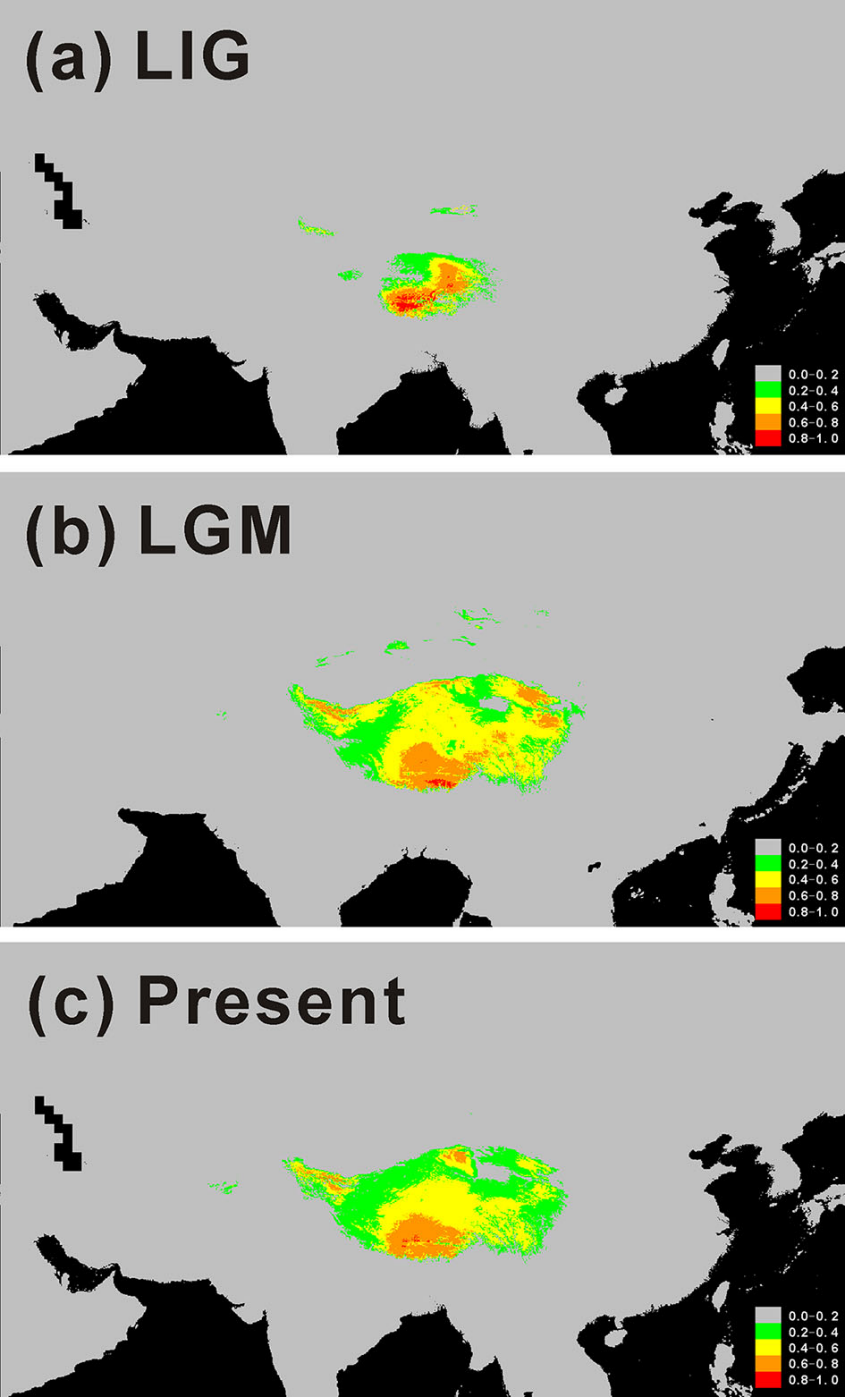
Potential distributions as probability of occurrence for *C. hendersonii* on the Tibetan Plateau. **(A)** At the Last Inter-Glacial (LIG; ~ 120 000–140 000 years before present); **(B)** At the Last Glacial Maximum (LGM; ~ 21 000 years before present); **(C)** Under current conditions (1950–2000).

In order to track historical distribution changes of *C. hendersonii*, we modeled the potential distribution for the LIG, LGM, and present (**Figure 5**). In the process of optimized variable selection, the model with the lowest AICc was built based on the following two uncorrelated environment variables with a model contribution >5.17% (**Table S4**): Min temperature of coldest month and Annual precipitation. The Annual precipitation was the most important variable (94.83% model contribution, **Table S4**) in discriminating suitable from nonsuitable habitats. Therefore, those two variables were used to conduct the following species distribution models. Paleo distribution models suggested that *C. hendersonii* continued its expansion during the LGM because the spatial distribution at the time of the LIG (**Figure 5A**) was predicted to be smaller than that during the LGM (**Figure 5B**). During the LIG, its distribution area was predicted to be small and limited to northeastern and central Tibet (see **Figure 5A**). Since the LGM, however, there has been little change in the predicted spatial distribution despite climate warming, and the simulated distribution based on present climate data was mostly congruent with the current ranges of this species (**Figure 5C**).

## Discussion

### Multiple Refugia and Demographic History of *C. hendersonii*

Several recent phylogeographic studies that focused on the flora endemic to the Tibetan Plateau, provided very different scenarios for responses of those species to past climatic oscillations (Qiu et al., 2011). Numerous tree species and other montane species currently occurring on the plateau platform experienced extent extinctions and/or population contraction and retreated to potential refugia located in the north- and/or south-eastern plateau with lower latitude/altitude during glacial periods. Subsequently, range expansions and recolonizations toward to higher latitude/altitude platform regions occurred during the following postglacial periods, thus showing higher genetic diversity in refugia regions and diversity decreases as the distance to the refugia increases (Zhang et al., 2005; Meng et al., 2007; Chen et al., 2008a; Yang et al., 2008; Cun and Wang, 2010; Wang et al., 2014; Zhang et al., 2018), mainly due to the founder effects. Another glacial “*in situ* survival” hypothesis revealed that some plant groups were capable of surviving *in situ* with range contraction at refugia of the southern plateau during past glacial periods, and the similar range expansions to the upper slopes did occur at the local scale during the interglacial or warm periods (Opgenoorth et al., 2009; Wu et al., 2010). In contrast, there is also molecular-based evidence that some alpine plants expanded its range on the Plateau during periods of climatic cooling and contracted to the interior platform during warm periods (e.g. Wang et al., 2009a; Li et al., 2010; Shimono et al., 2010; Sun et al., 2010; Jia et al., 2011; Jia et al., 2012), thus the interior region acted as a refugium during interglacial or warm periods and greatly contributed to the diversiﬁcation of such species. However, some cold-and drought-tolerant alpine plants, which only restricted to extremely high mountain areas on the Tibetan plateau, might have experienced a more complex demographic history.

In this study, we reconstructed the demographic history of the endemic alpine species of *C. hendersonii* on the plateau by using genetic variation data and species distribution modeling. Population genetic variation evidence suggested that the central, as well as the northeastern region of QTP, may have served as refugia for *C. hendersonii*. The cpDNA haplotype distributions of the central populations (sites 6–13) exhibited high levels of genetic diversity (**Table 1**), and unique ancestral haplotypes (C6, C7, and C8) were generally dominant and restricted to small areas within these populations (**Figures 1A, C**). Particularly, populations 6 and 8 seemed to be the longest surviving population in the sampling area of this study, because they not only showed the highest genetic diversity among all central populations (**Table 1**), but also harbored the ancestral haplotypes (C6, C7, and C8) and one widespread haplotype (C3) which was fixed in most populations in the southwestern and western QTP at high frequencies (**Figures 1A, C**). In addition, populations 1 and 2 in northeastern Tibet harbored three unique ancestral haplotypes (C1, C2, and C4) which were not found in other populations, although the widespread haplotype C3 was found at low frequencies in population 1 (**Figure 1C**). Meanwhile, the ITS haplotype distributions of *C. hendersonii* suggested similar genetic patterns within the central and northeastern Tibet. The central populations exhibited the highest genetic diversity (**Table S10**), and the most of haplotypes (H4–H29) were fixed within these populations (**Figure 2B**). Two unique haplotypes (H1 and H2) were only occurred in northeastern populations and one ancestral haplotype H3 was found at high frequencies in this region, although the genetic diversity of the northeastern populations was the lowest among four genetic groups due to its limited numbers of haplotypes (**Table S10**). The interior region on the QTP may have served as a refugium was also revealed in other alpine plant species, such as herbs (Wang et al., 2009a; Gao et al., 2012), shrubs (Wang et al., 2009b; Shimono et al., 2010; Jia et al., 2011; Zhang et al., 2012), and in cold-tolerant trees (Opgenoorth et al., 2009). However, the central and northeastern region probably provided a suitable habitat for *C. hendersonii* during interglacial period rather than during glacial period, because the species distribution modeling results observed rang constriction during LIG and massive range expansion during LGM (see **Figure 4B**).

Our results strongly suggested that populations of *C. hendersonii* experienced drastic range expansions in response to past cooling. Firstly, the large numbers of private cpDNA haplotypes (C9–C15) together with the star-like phylogeny patterns radiating from one dominant haplotype C3 (**Figure 1B**) were detected in this study, which are usually indicative of historical range expansion (Slatkin and Hudson, 1991). The widespread occurrences of these haplotypes on the southwestern and western Tibet (**Figure 1C**), combined with relatively low genetic diversity within populations (**Table 2**), further indicated that population expansions did occur from restricted source areas, such as the central and/or the northeastern regions of the QTP. Secondly, our ITS haplotype distribution showed that two dominant haplotypes (H12 and H20) were widespread in the populations of the southwestern plateau at high frequency (**Figure 2B**), suggesting that there might be population expansion in this region during the past climatic oscillations. Thirdly, such expansions were also supported by the results of demographic test, including mismatch distribution and EBSP analyses (**Figure 4**), suggesting that southwestern and western populations of *C. hendersonii* might have experienced recently population expansions. Time estimates dated the range expansion of southwestern populations to approximately between 122 and 14 Kya (**Table 5**) (around 45 to 30 Kya based on EBSP analysis, **Figure 4B**), thus after the termination of Eemian (starting around 126 kya), while the expansion of western populations occurred more recently (between 27 and 3 Kya, **Table 5**; around 15 Kya based on EBSP analysis, **Figure 4B**), likely during the last glacial maximum (LGM, starting around 20 Kya). Although these estimates were based on a range of possible mutation rates due to lack of fossil records, the time range was consistent with the timescale estimated by previous studies that focused on other alpine species in the same area (e.g. Shimono et al., 2010; Sun et al., 2010), and our results of species distribution modeling provided further support for these conclusions, suggesting that *C. hendersonii* continued its expansion during the LGM (**Figure 5**).

The evidence for these recent range expansion revealed that the climatic oscillations of LGM and following it may have caused a major shift in the range of this species in the southwestern and western Tibet. However, central and northeastern populations most likely did not experience signiﬁcant population size changes (**Figure 4** and **Table 5**). The demographic history of *C. hendersonii* described here are inconsistent with phylogeographic histories of other alpine plants on the Tibetan Plateau (e.g. Yang et al., 2008; Wang et al., 2009a; Shimono et al., 2010; Sun et al., 2010). For instance, phylogeographic studies on one alpine shrub species *Potentilla fruticosa*, which has relatively similar distribution areas with *C. hendersonii*, suggested that this species may have radically expanded across the whole Plateau before the LGM, besides the possible recently postglacial expansion from the central plateau to the northeastern plateau (Shimono et al., 2010; Sun et al., 2010). But we did not detect corresponding genetic signature of early range expansion for *C. hendersonii*, and it might have survived on northeastern as well as central Tibetan Plateau for a longtime during past interglacial and glacial periods. A similar demographic pattern was also reported for *Pomatosace ﬁlicula* (Wang et al., 2014), a small-sized herbaceous alpine plant, having almost the same distribution range and elevations as the northeastern and central populations of *C. hendersonii*.

### Genetic Diversiﬁcation

The complex local topography as well as drastic climate oscillations during past glacial and interglacial periods on the Tibetan Plateau have facilitated inter- and intra-specific diversification (Sun, 2002a; Sun, 2002b), and thus shaped distinct geographic genetic structure patterns in many plant species (Cun and Wang, 2010; Qiu et al., 2011; Liu et al., 2012; Liu et al., 2014; Wen et al., 2014). Our genetic survey across 30 population of *C. hendersonii* revealed a unique genetic structure. Based on cpDNA dataset, AMOVA analyses detected high genetic differentiation through the whole sampling range (*F*_ST_ = 0.877), and a distinct phylogeographic structure was detected among populations in *C. hendersonii* (*N*_ST_ = 0.894, *G*_ST_ = 0.854, P < 0.05). Those results were consistent with previous studies on alpine taxa which suggested that high genetic differentiation among populations within a species was usually coupled with the obvious population structure (Avise, 2004; Zhang et al., 2005; Chen et al., 2008b; Yang et al., 2008; Yang et al., 2012). *F*st analysis further suggested that populations from central Tibet, appeared to be the most strongly differentiated lineage, with *F*_ST_ values as high as 0.4654 and 0.3985 (**Table 4**) with respect to southwestern and western populations, and later two population groups showed relatively lower total gene diversity and genetic differentiation within populations (**Tables 2** and **4**), possibly due to the recent expansion and founder effects. Based on ITS DNA dataset, although AMOVA analyses detected a relatively lower genetic differentiation through the whole sampling range (*F*_ST_ = 0.283) compared to that suggested by cpDNA dataset, a distinct phylogeographic structure was also detected among populations in *C. hendersonii* (*N*_ST_ = 0.359, *G*_ST_ = 0.219, P < 0.05). *F*st analysis also showed high intraspecific divergences between each pair of the four genetic groups (**Table 4**). Thus, it should be clear that the range expansion mentioned above may have greatly contributed to the deep intraspecific diversification in *C. hendersonii*.

However, population genetic structure analysis based on the ITS sequence variations revealed a little different phylogeographic pattern for *C. hendersonii*. The western populations shared the same nuclear (ITS) gene pool with those from other regions, especially from the populations of northeastern Tibet, showing a distinct signal of genetic admixture (**Figure 3**). Such a pattern was further revealed by the distribution and relationship of the ITS haplotypes. One widespread haplotype (H3) was mainly shared by both northeastern and western populations (**Figure 2B**); the majority of haplotypes (H41–H48) only found in the western populations showed more close phylogenetic relationships with that (H1–H3) fixed by the northeastern populations than any others (**Figure 2** and **Figure S1**). Those pieces of evidence might suggest that the expansion and colonization of the western populations most likely occurred by the long-distance dispersal from more than one restricted source area. On the other hand, based on the species-speciﬁc variation comparisons between ITS and cpDNA, we found that interspeciﬁc lineage sorting at nuclear ITS may be much faster than that at cpDNA in *C. hendersonii*. For example, the distribution of the ITS variations between central and northeastern populations, as well as between southwestern and western populations, showed a more clear geographic pattern than that of cpDNA (**Figures 1C**, **2B**, and **3B**). In fact, this was also recorded to be the case within the intraspeciﬁc studies (Wang et al., 2009b). This was probably due to their different inheritance modes (maternal *vs.* bipaternal) and dispersing style (seeds *vs.* both pollen and seeds) among populations, as well as the fast substitution rate and the concerted evolution of the ITS sequences (Alvarez and Wendel, 2003). Therefore, if ITS variation is found to be variable within a single species, it may be highly helpful to combine two different genetic markers when doing phylogeographic studies. This may give more weight to inferences on genetic divergences and introgressions between and within species (Liu et al., 2012).

## Data Availability Statement

The datasets generated for this study can be found in the GenBank under accession numbers MT023736–MT023784.

## Author Contributions

JZ and QL conceived and designed the research. XG and XL performed the experiments. JZ, JN, and DD collected and analyzed the data. JZ, QL, and LO wrote and revised the paper. All authors read and approved the final manuscript.

## Funding

This research was supported by the National Natural Science Foundation of China (31860585 and 41761009), the Natural Science Foundation of Qinghai Science & Technology Department (2019-ZJ-907), the State key laboratory construction project (2015DQ870717), the Open Project of State Key Laboratory of Plateau Ecology and Agriculture, Qinghai University (2017-ZZ-03), the Natural Science Basic Research Plan in Shaanxi Province of China (2019JQ-051 and 2019SF-307), and the Fundamental Research Funds for the Central Universities (GK201703034).

## Conflict of Interest

The authors declare that the research was conducted in the absence of any commercial or financial relationships that could be construed as a potential conflict of interest.
