# Supplementary material for: Molecular Phylogeography and Evolutionary History of the Endemic Species Corydalis hendersonii (Papaveraceae) on the Tibetan Plateau Inferred From Chloroplast DNA and ITS Sequence Variation
Source: Front Plant Sci. 2020 Apr 8;11:436. doi: 10.3389/fpls.2020.00436 (PMC7160248; doi:10.3389/fpls.2020.00436)
Supplement: Supplementary file 1 [file DataSheet_1.docx]

TABLE S1 | The primer sequences for PCR amplification.

| Region | Primer sequences | Reference |
| --- | --- | --- |
| *trn*S-*trn*G | GCCGCTTTAGTCCACTCAGC | Hamilton, 1999 |
|  | GAACGAATCACACTTTTACCAC |  |
| *trn*T-*trn*L | CATTACAAATGCGATGCTCT | Taberlet et al., 1991 |
|  | TCTACCGATTTCGCCATATC |  |
| *atp*H-*atp*I | CCAGCAGCAATAACGGAAGC | Grivet et al., 2001 |
|  | ATAGGTGAATCCATGGAGGG |  |
| *psb*E-*pet*L | f-ATCTACTAAATTCATCGAGTTGTTCC | Dong et al., 2012 |
|  | r-TATCTTGCTCAGACCAATAAATAGA |  |
| ITS | ITS1-TCCGTAGGTGAACCTGCGG | Gaskin and Wilson, 2007 |
|  | ITS4-TCCTCCGCTTATTGATATGC |  |

TABLE S2 | PCR programs employed for each primer pair.

|  |  | 37 Cycles | | |  |
| --- | --- | --- | --- | --- | --- |
| Primer pair | First denaturing | Denaturing | Annealing | Extension | Final extension |
| *trn*S-*trn*G | 94℃, 5m | 94℃, 45s | 59℃, 40s | 72℃, 1m | 72℃, 7m |
| *trn*T-*trn*L | 94℃, 5m | 94℃,1m | 53℃, 1m | 72℃,1m, | 72℃, 7m |
| *atp*H-*atp*I | 95℃, 5m | 95℃,39s | 52℃, 30s | 72℃, 1m30s | 72℃, 5m |
| *psb*E-*pet*L | 95℃, 5m | 95℃, 30s | 52℃, 30s | 72℃, 1m30s | 72℃, 5m |
| ITS | 95℃, 5m | 95℃, 30s | 61℃, 30s | 72℃, 1m30s | 72℃, 5m |

TABLE S3 | Localities of museum records used for niche modeling.

| Population code | Latitude (°N) | Longitude (°E) | Altitude (m) |
| --- | --- | --- | --- |
| pop1 | 31.56 | 97.67 | 5422 |
| pop2 | 31.21 | 96.60 | 4864 |
| pop3 | 31.88 | 94.49 | 4912 |
| pop4 | 31.48 | 93.68 | 5774 |
| pop5 | 30.75 | 93.09 | 5224 |
| pop6 | 29.66 | 92.45 | 5706 |
| pop7 | 29.83 | 92.35 | 4961 |
| pop8 | 30.42 | 92.21 | 5543 |
| pop9 | 32.87 | 91.93 | 5326 |
| pop10 | 30.61 | 91.93 | 5266 |
| pop11 | 28.71 | 91.80 | 5947 |
| pop12 | 28.35 | 91.77 | 5164 |
| pop13 | 28.80 | 91.77 | 5460 |
| pop14 | 28.97 | 91.32 | 5128 |
| pop15 | 30.12 | 91.27 | 5580 |
| pop16 | 29.75 | 91.21 | 5623 |
| pop17 | 28.63 | 91.19 | 5135 |
| pop18 | 30.69 | 91.11 | 5213 |
| pop19 | 28.99 | 91.09 | 4785 |
| pop20 | 29.17 | 90.23 | 4850 |
| pop21 | 28.89 | 90.19 | 4927 |
| pop22 | 29.63 | 89.47 | 5050 |
| pop23 | 29.46 | 89.04 | 5335 |
| pop24 | 29.46 | 88.40 | 5050 |
| pop25 | 29.05 | 86.59 | 4785 |
| pop26 | 32.75 | 87.65 | 5706 |
| pop27 | 31.77 | 84.94 | 5623 |
| pop28 | 32.38 | 82.32 | 5335 |
| pop29 | 31.03 | 81.29 | 5947 |
| pop30 | 31.66 | 80.12 | 5100 |
| pop31 | 33.42 | 86.71 | 5250 |
| pop32 | 30.47 | 91.10 | 5400 |
| pop33 | 35.72 | 89.31 | 4900 |
| pop34 | 31.17 | 89.63 | 5000 |
| pop35 | 31.67 | 90.09 | 4500 |
| pop36 | 30.93 | 88.71 | 5100 |
| pop37 | 33.69 | 87.98 | 5000 |
| pop38 | 31.76 | 88.37 | 5300 |
| pop39 | 29.68 | 89.10 | 4950 |
| pop40 | 28.66 | 87.13 | 5400 |
| pop41 | 28.90 | 85.39 | 5070 |
| pop42 | 28.63 | 85.27 | 4700 |
| pop43 | 30.29 | 81.18 | 5000 |
| pop44 | 33.25 | 80.23 | 5100 |
| pop45 | 34.23 | 80.60 | 5200 |
| pop46 | 29.82 | 90.68 | 5500 |
| pop47 | 30.47 | 91.10 | 5300 |
| pop48 | 34.32 | 95.32 | 5200 |
| pop49 | 34.01 | 92.10 | 4700 |
| pop50 | 35.10 | 90.57 | 4800 |
| pop51 | 35.34 | 92.35 | 5100 |
| pop52 | 34.52 | 93.03 | 4750 |
| pop53 | 34.27 | 90.41 | 4900 |
| pop54 | 33.10 | 94.23 | 5100 |
| pop55 | 34.81 | 80.06 | 4900 |
| pop56 | 37.64 | 77.37 | 4750 |
| pop57 | 36.54 | 76.44 | 4500 |
| pop58 | 36.32 | 77.41 | 4700 |
| pop59 | 36.83 | 78.20 | 4800 |
| pop60 | 37.05 | 75.43 | 5000 |

TABLE S4 | Variable selection process for models with a beta-multiplier of 5.5. In step one, we compiled an initial MAXENT model (model 1) with all 19 variables and excluded 17 variables due to relative model contribution scores < 5%. In step two, the remaining set of variables was used to compile a new MAXENT model (model 2). The variables and their contributions in the model of highest performance (lowest AICc, model 2) were marked in yellow. Numbers in red indicate the exclusion of variables from one step to the next. AUC.Test, the area under the receiver operating characteristic estimated from test data (maximum test AUC); AUC.Diff, the difference between AUC values estimated from test and training data.

|  | Model 1 |  | Model 2 |  |
| --- | --- | --- | --- | --- |
| Bioclimatic variables | Contributions | Correlation coefficients | Contributions | Correlation coefficients |
| Bio1 Annual Mean Temperature | 0 | NA | NA | NA |
| Bio2 Mean Diurnal Range (Mean of monthly (max temp - min temp)) | 0 | NA | NA | NA |
| Bio3 Isothermality (BIO2/BIO7) (* 100) | 0 | NA | NA | NA |
| Bio4 Temperature Seasonality (standard deviation *100) | 0 | NA | NA | NA |
| Bio5 Max Temperature of Warmest Month | 0 | NA | NA | NA |
| Bio6 Min Temperature of Coldest Month | 12.3755 | -0.09219 | 5.1702 | 1 |
| Bio7 Temperature Annual Range (BIO5-BIO6) | 0.0003 | NA | NA | NA |
| Bio8 Mean Temperature of Wettest Quarter | 0 | NA | NA | NA |
| Bio9 Mean Temperature of Driest Quarter | 0 | NA | NA | NA |
| Bio10 Mean Temperature of Warmest Quarter | 0 | NA | NA | NA |
| Bio11 Mean Temperature of Coldest Quarter | 0 | NA | NA | NA |
| Bio12 Annual Precipitation | 86.9996 | 1 | 94.8298 | -0.09219 |
| Bio13 Precipitation of Wettest Month | 0.6247 | NA | NA | NA |
| Bio14 Precipitation of Driest Month | 0 | NA | NA | NA |
| Bio15 Precipitation Seasonality (Coefficient of Variation) | 0 | NA | NA | NA |
| Bio16 Precipitation of Wettest Quarter | 0 | NA | NA | NA |
| Bio17 Precipitation of Driest Quarter | 0 | NA | NA | NA |
| Bio18 Precipitation of Warmest Quarter | 0 | NA | NA | NA |
| Bio19 Precipitation of Coldest Quarter | 0 | NA | NA | NA |
| AICc | 2913.026 |  | 2903.653 |  |
| AUC.Test | 0.9976 |  | 0.9979 |  |
| AUC.Diff | 0.0005 |  | 0.0001 |  |

TABLE S5 | *F_CT_* values for different numbers of population groups (*K*) inferred by the SAMOVA algorithm using the cpDNA dataset.

| *K* | Population grouping | *F*_CT_ | *P*_value |
| --- | --- | --- | --- |
| *K* = 2 | (1, 2, 3, 4, 5, 6, 12, 13, 14, 15, 17, 18, 19, 20, 21, 22, 23, 24, 25, 26, 27, 28, 29, 30) (7, 8, 9, 10, 11, 16) | 0.74347 | 0.00000 |
| *K* = 3 | (3, 4, 5, 6, 12, 13, 14, 15, 17, 18, 19, 20, 21, 22, 23, 24, 25, 26, 27, 28, 29, 30) (7, 8, 9, 10, 11, 16) (1, 2) | 0.80544 | 0.00000 |
| *K* = 4 | (3, 4, 5, 6, 12, 13, 14, 15, 17, 18, 19, 20, 21, 22, 23, 24, 25, 26, 27, 28, 29, 30) (9, 10, 11) (7, 8, 16) (1, 2) | 0.84771 | 0.00000 |
| *K* = 5 | (3, 4, 5, 6, 12, 13, 14, 15, 17, 18, 19, 20, 21, 22, 23, 24, 25, 26, 27, 28, 29, 30) (9, 10, 11) (7, 8, 16) (1, 2) (5) | 0.87323 | 0.00000 |
| *K* = 6 | (3, 4, 5, 6, 12, 13, 14, 15, 17, 18, 19, 20, 21, 22, 23, 24, 25, 26, 27, 28, 29) (9, 10, 11) (1, 2) (7, 8) (16) (30) | 0.86642 | 0.00000 |
| *K* = 7 | (3, 4, 6, 12, 14, 15, 17, 18, 19, 20, 21, 22, 23, 24, 25, 26, 27, 28, 29) (9, 10, 11) (1, 2) (7, 8, 16) (13) (30) (5) | 0.92107 | 0.00000 |
| *K* = 8 | (3, 4, 6, 12, 14, 15, 17, 18, 19, 20, 21, 22, 24, 25, 26, 27, 28, 29) (9, 10, 11) (1, 2) (7, 8, 16) (13) (30) (5) (23) | 0.91706 | 0.00000 |
| *K* = 9 | (3, 4, 6, 12, 14, 15, 17, 18, 19, 20, 21, 22, 24, 25, 26, 27, 28, 29) (9, 10, 11) (7, 8, 16) (13) (30) (5) (23) (1) (2) | 0.94320 | 0.00000 |
| *K* = 10 | (12, 13, 14, 15, 17, 18, 19, 20, 21, 22, 23, 24, 25, 26, 27, 28, 29) (3, 4, 6) (9, 10) (7, 8) (16) (30) (5) (1) (2) (11) | 0.91465 | 0.00000 |
| *K* = 11 | (12, 14, 15, 17, 18, 19, 20, 21, 22, 23, 24, 25, 26, 27, 28, 29) (3, 4, 6) (7, 8, 16) (9) (10) (30) (5) (1) (2) (11) (13) | 0.93833 | 0.00000 |
| *K* = 12 | (12, 14, 17, 18, 19, 21, 22, 23, 24, 25, 26, 27, 28, 29) (3, 4, 6) (7, 8, 16) (9, 10) (30) (5) (1) (2) (11) (13) (15) (20) | 0.93509 | 0.00000 |
| *K* = 13 | (12, 14, 17, 18, 19, 20, 21, 22, 23, 24, 25, 26, 27, 28, 29) (10, 11) (3, 4, 6) (7) (8) (16) (9) (30) (5) (1) (2) (13) (15) | 0.93935 | 0.00000 |
| *K* = 14 | (4, 6, 12, 14, 17, 18, 19, 21, 22, 23, 24, 25, 26, 27, 28, 29) (10, 11) (7) (8) (16) (9) (30) (5) (1) (2) (13) (15) (3) (20) | 0.93931 | 0.00000 |
| *K* = 15 | (14, 17, 18, 19, 21, 22, 23, 24, 25, 26, 27, 28, 29) (3, 4) (10, 11) (7, 8) (16) (9) (30) (5) (1) (2) (13) (15) (20) (6) (12) | 0.93598 | 0.00000 |
| *K* = 16 | (12, 14, 17, 18, 19, 21, 22, 24, 25, 27, 28, 29) (3, 4, 6) (9, 10) (7) (8) (16) (30) (5) (1) (2) (11) (13) (15) (20) (23) (26) | 0.93482 | 0.00000 |

TABLE S6 | *F_CT_* values for different numbers of population groups (K) inferred by the SAMOVA algorithm using the ITS dataset.

| *K* | Population grouping | *F*_CT_ | *P*-value |
| --- | --- | --- | --- |
| *K* = 2 | (1, 2, 3, 4, 5, 27, 29, 30) (6, 7, 8, 9, 10, 11, 12, 13, 14, 15, 16, 17, 18, 19, 20, 21, 22, 23, 24, 25, 26, 28) | 0.28906 | 0.00000 |
| *K* = 3 | (1, 2, 3, 4, 5, 27, 29, 30) (6, 7, 8, 9, 10, 11, 12, 13) (14, 15, 16, 17, 18, 19, 20, 21, 22, 23, 24, 25, 26, 28) | 0.31683 | 0.00000 |
| *K* = 4 | (1, 2, 3, 4, 5) (6, 7, 8, 9, 10, 11, 12, 13) (14, 15, 16, 17, 18, 19, 20, 21, 22, 23, 24, 25, 26) (27, 28, 29, 30) | 0.33051 | 0.00000 |
| *K* = 5 | (1, 2, 3, 4, 5) (6, 7, 8, 9, 10, 11, 12, 13) (14, 15, 16, 17, 18, 19, 20, 21, 22, 23, 25, 26, 28) (27, 29, 30) (24) | 0.32990 | 0.00000 |
| *K* = 6 | (1, 2, 3, 4, 5, 27, 29, 30) (6, 7, 8, 9, 10, 11, 12) (14, 15, 16, 17, 18, 19, 20, 21, 22, 23, 25, 26) (13) (24) (28) | 0.36727 | 0.00000 |
| *K* = 7 | (1, 2, 3, 4, 5) (6, 7, 8, 9, 10, 11, 12) (14, 15, 16, 17, 18, 19, 20, 21, 22, 23, 25, 26) (27, 29, 30) (13) (24) (28) | 0.36701 | 0.00000 |
| *K* = 8 | (1, 2, 3, 4, 5) (6, 7, 8, 9, 10, 11, 12) (14, 15, 16, 17, 18, 19, 20, 21, 22, 23, 26) (27, 29, 30) (13) (24) (25) (28) | 0.36852 | 0.00000 |
| *K* = 9 | (1, 2, 3, 4, 5) (6, 9, 10, 11) (14, 15, 16, 17, 18, 19, 20, 21, 22, 23, 25, 26) (27, 29, 30) (7, 8) (13) (12) (24) (28) | 0.39223 | 0.00000 |
| *K* = 10 | (1, 2, 3, 4) (6, 7, 8, 9, 10, 11) (14, 15, 16, 17, 18, 19, 20, 21, 22, 23, 26) (27, 29, 30) (5) (13) (12) (24) (25) (28) | 0.39597 | 0.00000 |
| *K* = 11 | (1, 2, 3, 4, 5) (6, 9, 10, 11) (14, 15, 16, 17, 18, 19, 20, 21, 26) (27, 29, 30) (7, 8) (22, 23) (13) (12) (24) (25) (28) | 0.40138 | 0.00000 |
| *K* = 12 | (1, 2, 3, 4) (6, 9, 10, 11) (14, 15, 16, 17, 18, 19, 20, 21, 26) (27, 29, 30) (7, 8) (22, 23) (5) (13) (12) (24) (25) (28) | 0.41349 | 0.00000 |
| *K* = 13 | (2, 3, 4) (6, 9, 10, 11) (15, 16, 17, 18, 19, 20, 21, 26) (27, 29, 30) (7, 8) (22, 23, 24) (1) (5) (13) (12) (14) (25) (28) | 0.39686 | 0.00000 |
| *K* = 14 | (2, 3, 4) (6, 9, 10, 11) (14, 15, 16, 17, 18, 19, 20, 21, 26) (27, 29) (7, 8) (22, 23) (1) (5) (13) (12) (24) (25) (28) (30) | 0.41693 | 0.00000 |
| *K* = 15 | (1, 2, 3, 4) (6, 9, 10, 11) (14, 15, 16, 17, 18, 20, 21) (27, 29) (7, 8) (22, 23) (5) (13) (12) (19) (24) (25) (26) (28) (30) | 0.40401 | 0.00000 |
| *K* = 16 | (2, 3, 4) (6, 10) (9, 11) (14, 15, 16, 17, 18, 20, 21) (27, 29, 30) (7, 8) (22, 23) (1) (5) (13) (12) (19) (24) (25) (26) (28) | 0.40452 | 0.00000 |

TABLE S7 | Sequence lengths and variations of four chloroplast DNA markers.

| Region | Aligned length (bp) | No. of substitutions | No. of indels | No. of variable sites (%) |
| --- | --- | --- | --- | --- |
| *trn*S*-trn*G | 465 | 5 | 1 | 6（0.13） |
| *trn*T*-trn*L | 677 | 2 | 1 | 3（0.04） |
| *atp*H*-atp*I | 1151 | 0 | 3 | 3（0.03） |
| *psb*E*-pet*L | 975 | 1 | 0 | 1（0.01） |
| All four chloroplast regions | 3268 | 8 | 5 | 13（0.04） |

TABLE S8 | Variable nucleotide sites in each of the fifteen Chlorotypes detected in *C. hendersonii*.

| Chlorotype | *trn*S*-trn*G | | | | | | *trn*T*-trn*L | | | *atp*H*-atp*I | | | *psb*E*-pet*L |
| --- | --- | --- | --- | --- | --- | --- | --- | --- | --- | --- | --- | --- | --- |
|  | 40 | 263 | 303 | 313 | 357 | 376 | 53 | 67-71 | 393 | 495-499 | 798 | 941-945 | 956 |
| C1 | - | T | G | C | C | T | C | CATAT | G | ----- | A | ATAGC | G |
| C2 | - | T | G | C | C | T | C | CATAT | A | ----- | A | ATAGC | G |
| C3 | - | T | G | C | C | T | T | CATAT | A | ----- | A | ATAGC | G |
| C4 | - | T | G | C | C | T | C | CATAT | A | TTTAT | A | ATAGC | G |
| C5 | - | T | G | C | C | T | T | CATAT | A | ----- | A | ----- | G |
| C6 | - | T | G | C | G | T | T | CATAT | A | ----- | - | ATAGC | G |
| C7 | - | T | G | C | G | T | C | CATAT | A | ----- | - | ATAGC | G |
| C8 | - | T | G | C | C | T | T | CATAT | A | ----- | - | ATAGC | G |
| C9 | - | T | G | C | C | T | T | CATAT | A | ----- | A | ATAGC | A |
| C10 | - | T | G | C | C | T | T | ----- | A | ----- | A | ATAGC | G |
| C11 | - | T | G | T | C | T | T | CATAT | A | ----- | A | ATAGC | G |
| C12 | - | T | T | C | C | T | T | CATAT | A | ----- | A | ATAGC | G |
| C13 | - | C | G | C | C | T | T | CATAT | A | ----- | A | ATAGC | G |
| C14 | A | T | G | C | C | T | T | CATAT | A | ----- | A | ATAGC | G |
| C15 | - | T | G | C | C | C | T | CATAT | A | ----- | A | ATAGC | G |

TABLE S9 | Variable nucleotide sites in each of the forty-nine ITS haplotypes detected in *C. hendersonii*.

| Haplotypes | 23 | 24 | 48 | 65 | 72 | 87 | 130 | 330 | 331 | 339 | 347 | 363 | 372 | 384 | 396 | 497 | 473 | 476 | 495 | 499 | 503 | 504 | 509 | 511 | 536 |
| --- | --- | --- | --- | --- | --- | --- | --- | --- | --- | --- | --- | --- | --- | --- | --- | --- | --- | --- | --- | --- | --- | --- | --- | --- | --- |
| H1 | A | A | A | C | A | C | G | C | G | T | G | G | C | C | C | C | C | C | G | C | C | C | G | C | G |
| H2 | C | A | G | C | A | C | G | C | G | T | G | G | C | C | C | C | C | C | G | C | C | C | G | C | G |
| H3 | C | A | A | C | A | C | G | C | G | T | G | G | C | C | C | C | C | C | G | C | C | C | G | C | G |
| H4 | C | A | G | C | A | C | G | C | G | T | A | G | C | C | C | C | C | C | T | C | A | C | G | C | G |
| H5 | C | A | A | C | A | C | G | C | G | T | A | G | C | C | C | C | C | C | T | C | C | C | G | C | G |
| H6 | C | A | G | C | A | C | G | C | G | T | G | G | C | C | C | C | C | C | T | C | A | C | G | C | G |
| H7 | C | A | G | C | A | C | G | C | G | T | G | G | C | C | C | C | C | C | T | C | C | C | G | C | G |
| H8 | C | A | A | C | A | C | G | C | G | T | A | G | C | C | C | C | C | C | T | C | A | C | G | C | G |
| H9 | C | A | A | C | A | C | G | C | G | T | A | A | C | C | C | C | C | C | T | C | C | C | G | C | G |
| H10 | C | A | A | C | A | C | G | C | A | T | A | G | C | C | C | C | C | C | T | C | A | C | G | C | G |
| H11 | C | A | G | C | A | C | G | C | G | T | A | G | C | C | C | C | C | C | T | C | A | C | G | C | A |
| H12 | C | A | A | C | A | C | G | C | G | T | A | G | C | C | C | C | C | C | G | C | C | C | G | C | G |
| H13 | C | A | A | C | A | C | G | C | G | T | A | A | C | C | C | C | C | C | G | C | C | C | G | C | G |
| H14 | C | A | A | C | A | C | G | C | G | T | A | A | C | C | C | C | C | C | T | C | C | C | G | C | A |
| H15 | C | A | G | C | A | C | G | C | G | T | A | A | C | C | C | C | C | C | T | C | C | C | G | C | A |
| H16 | C | A | A | C | A | C | G | C | G | T | G | G | C | T | C | C | C | C | T | C | A | C | G | C | G |
| H17 | C | A | A | C | A | C | G | C | G | T | A | G | C | T | C | C | C | C | T | C | A | C | G | C | G |
| H18 | C | A | A | C | A | C | G | C | G | T | A | G | C | T | C | C | C | C | G | C | C | C | G | C | G |
| H19 | C | A | A | C | A | C | G | C | G | T | G | G | C | T | C | C | C | C | G | C | C | C | G | C | G |
| H20 | C | A | A | G | A | C | G | C | G | T | A | G | C | T | C | C | C | C | T | C | C | C | G | C | G |
| H21 | C | A | A | G | A | C | G | C | G | T | A | G | C | C | C | C | C | C | G | C | C | C | G | C | G |
| H22 | C | A | A | C | A | G | T | C | G | T | G | G | C | T | C | C | C | C | G | C | C | C | G | C | G |
| H23 | C | A | A | C | A | C | G | C | G | T | A | A | C | T | C | C | C | C | G | C | C | C | G | C | G |
| H24 | C | A | A | C | A | C | T | C | G | T | G | G | C | C | C | C | C | C | G | C | C | C | G | C | G |
| H25 | C | A | G | C | A | C | G | C | G | T | A | G | C | C | C | C | C | C | T | C | A | C | T | C | G |
| H26 | C | A | A | C | A | C | G | C | G | T | A | G | C | C | C | C | C | C | A | C | C | C | G | C | G |
| H27 | C | A | G | C | A | C | G | T | G | T | A | G | C | T | T | T | T | C | A | T | C | C | G | C | G |
| H28 | C | A | A | C | A | C | G | C | G | T | A | A | C | C | C | C | C | C | A | C | C | C | G | C | G |
| H29 | C | A | A | C | A | C | G | C | G | T | A | G | C | T | C | C | C | C | T | C | C | C | G | C | G |
| H30 | C | A | G | C | A | C | G | C | G | T | A | G | C | C | C | C | C | C | T | C | C | C | G | C | G |
| H31 | C | A | A | C | A | C | T | C | G | T | A | G | C | C | C | C | C | C | G | C | C | C | G | C | G |
| H32 | C | A | A | G | A | C | G | C | G | T | G | G | C | T | C | C | C | C | T | C | C | C | G | T | G |
| H33 | C | A | A | C | A | C | T | C | G | C | G | G | C | C | C | C | C | C | G | C | C | C | G | C | G |
| H34 | C | A | A | C | A | C | G | C | G | C | G | G | C | T | C | C | C | C | G | C | C | C | G | C | G |
| H35 | C | A | A | C | A | C | G | C | G | C | A | A | C | C | C | C | C | C | T | C | C | C | G | C | G |
| H36 | C | A | A | C | A | C | G | C | G | C | A | G | C | C | C | C | C | C | G | C | C | C | G | C | G |
| H37 | C | A | A | C | A | C | G | C | G | C | A | G | C | T | C | C | C | C | G | C | C | C | G | C | G |
| H38 | C | A | A | C | A | C | G | C | G | T | G | A | C | C | C | C | C | C | G | C | C | C | G | C | G |
| H39 | C | A | A | T | A | C | G | C | G | T | G | G | C | T | C | C | C | C | T | C | C | C | G | C | G |
| H40 | C | A | A | T | A | C | G | C | G | T | A | G | C | T | C | C | C | C | T | C | C | C | G | C | G |
| H41 | C | A | A | C | A | C | G | C | G | T | G | G | C | C | C | C | C | C | G | C | C | T | G | C | G |
| H42 | C | A | A | C | A | C | G | C | G | T | A | G | C | C | C | C | C | C | G | C | C | T | G | C | G |
| H43 | C | A | A | C | A | C | G | C | G | T | G | G | C | T | C | C | C | T | G | C | C | T | G | C | G |
| H44 | C | A | A | C | A | C | G | C | G | T | A | G | A | T | C | C | C | T | G | C | C | T | G | C | G |
| H45 | C | A | A | C | A | C | G | C | G | T | A | G | C | T | C | C | C | C | G | C | C | T | G | C | G |
| H46 | C | A | A | C | A | C | G | C | G | T | A | G | C | T | C | C | C | T | G | C | C | T | G | C | G |
| H47 | C | A | A | C | G | C | G | C | G | T | G | G | C | C | C | C | C | C | G | C | C | C | G | C | G |
| H48 | C | G | A | C | A | C | G | C | G | T | G | G | T | C | C | C | C | C | G | C | C | C | G | C | G |
| H49 | C | A | A | C | A | C | G | C | A | T | G | G | C | T | C | C | C | C | G | C | C | C | G | C | G |

TABLE S10 | Sampling sites, sample size (*N*), ITS haplotypes distribution, estimates of haplotype diversity (*H*_E_) and nucleotide diversity (*π*) in 30 populations of *C. hendersonii*.

| Population code | Sample location | Latitude | Longitude | Altitude | Haplotypes ( frequencies ) | *H*_E_ | *π* × 10^-3^ | *N* |
| --- | --- | --- | --- | --- | --- | --- | --- | --- |
|  |  | (°N) | (°E) | (m) |  |  |  |  |
| Northeastern populations |  |  |  |  |  |  |  |  |
| 1 | Chamdo | 31.56 | 97.67 | 4704 | H1(3), H2(3) | 0.600 | 2.05 | 3 |
| 2 | Dengqen | 31.21 | 95.60 | 4830 | H1(1), H2(6), H3(11) | 0.542 | 1.00 | 9 |
| 3 | Sog | 31.88 | 94.49 | 4925 | H2(2), H3(2) | 0.667 | 1.14 | 2 |
| 4 | Biru | 31.48 | 93.68 | 4800 | H2(1), H3(5) | 0.333 | 0.57 | 3 |
| 5 | Amdo | 32.87 | 91.93 | 5623 | H1(10), H3(2), H12(2) | 0.484 | 1.20 | 7 |
| Average |  |  |  |  |  |  | 1.70 |  |
| Central populations |  |  |  |  |  |  |  |  |
| 6 | Lhari | 30.75 | 93.09 | 5335 | H3(1), H4(1), H5(3), H6(3), H7(1), H8(3) | 0.864 | 3.01 | 6 |
| 7 | Sangri | 29.66 | 92.45 | 4864 | H5(1), H8(2), H9(1), H10(4), H11(1), H12(1),  H13(1), 14(1) | 0.894 | 3.76 | 6 |
| 8 | Maldrogongkar | 29.83 | 92.35 | 4927 | H7(1), H8(1), H9(1), H10(3), H15(1), H16(1) | 0.893 | 4.89 | 4 |
| 9 | Maldrogongkar | 30.42 | 92.21 | 4969 | H3(5), H4(4), H17(1) | 0.644 | 4.11 | 5 |
| 10 | Nagchu | 30.61 | 91.93 | 4850 | H4(2), H5(2), H6(2), H8(2), H11(2), H12(1), | 0.934 | 4.38 | 7 |
|  |  |  |  |  | H18(1), H19(2) |  |  |  |
| 11 | Damxung | 30.69 | 91.11 | 5050 | H3(4), H4(3), H8(1), H19(1), H29(1) | 0.800 | 4.22 | 5 |
| 12 | Lhunzhub | 30.12 | 91.27 | 4785 | H9(1), H11(1), H15(1), H22(4), H23(1), H24(1),  H25(1) | 0.867 | 7.84 | 5 |
| 13 | Lhasa | 29.75 | 91.21 | 4912 | H26(2), H27(3), H28(1) | 0.733 | 7.76 | 3 |
| Average |  |  |  |  |  |  | 4.56 |  |
| Southwestern populations |  |  |  |  |  |  |  |  |
| 14 | Zhanang | 28.97 | 91.32 | 5135 | H12(3), H20(5), H21(2) | 0.689 | 2.70 | 5 |
| 15 | Comai | 28.80 | 91.77 | 4961 | H12(5), H20(5) | 0.556 | 2.85 | 5 |
| 16 | Comai | 28.71 | 91.80 | 5164 | H12(5), H20(5) | 0.556 | 2.85 | 5 |
| 17 | Comai | 28.35 | 91.77 | 5213 | H12(6), H20(6) | 0.545 | 2.80 | 6 |
| 18 | Gonggar | 28.99 | 91.09 | 5128 | H5(1), H12(4), H20(5) | 0.644 | 2.82 | 5 |
| 19 | Rinbung | 29.17 | 90.23 | 5266 | H12(1), H20(4), H24(1), H31(2), H32(2) | 0.822 | 4.95 | 5 |
| 20 | Gyangze | 28.89 | 90.19 | 5224 | H5(2), H12(2), H20(4) | 0.714 | 2.69 | 4 |
| 21 | Nagarze | 28.63 | 91.19 | 5422 | H12(2), H20(4), H21(1), H29(1), H30(2) | 0.822 | 3.31 | 5 |
| 22 | Namling | 29.63 | 89.47 | 5326 | H12(1), H18(1), H20(5), H24(2), H33(2), | 0.868 | 5.36 | 7 |
|  |  |  |  |  | H34(1), H35(1), H36(1) |  |  |  |
| 23 | Shigatse | 29.46 | 89.04 | 4884 | H3(1), H5(1), H9(1), H12(1), H19(1), H34(1) | 1.000 | 4.00 | 3 |
| 24 | Xaitongmoin | 29.46 | 88.40 | 5706 | H18(1), H23(2), H34(1), H36(2), H37(4) | 0.822 | 2.36 | 5 |
| 25 | Ngamring | 29.05 | 86.59 | 5774 | H3(1), H19(1), H23(1), H37(2), H38(1), H39(2), | 0.879 | 4.33 | 6 |
|  |  |  |  |  | H40(4) |  |  |  |
| 26 | Nyima | 32.75 | 87.65 | 5396 | H12(19), H20(19) | 0.514 | 2.64 | 19 |
| Average |  |  |  |  |  |  | 3.43 |  |
| Western populations |  |  |  |  |  |  |  |  |
| 27 | Gerze | 31.77 | 84.94 | 5580 | H3(3), H19(1), H26(2), H29(2), H41(1), H42(1) | 0.889 | 3.42 | 5 |
| 28 | Gegyai | 32.38 | 82.32 | 5947 | H12(1), H29(1), H41(2), H43(2), H44(5), | 0.857 | 3.82 | 7 |
|  |  |  |  |  | H45(2), H46(1) |  |  |  |
| 29 | Burang | 31.03 | 81.29 | 5543 | H3(4), H47(2), H48(2) | 0.714 | 2.20 | 4 |
| 30 | Gar | 31.66 | 80.12 | 5460 | H3(5), H43(1), H44(1), H49(3) | 0.788 | 3.53 | 5 |
| Average |  |  |  |  |  |  | 3.24 |  |
| Total |  |  |  |  |  | 0.915 | 5.11 | 166 |


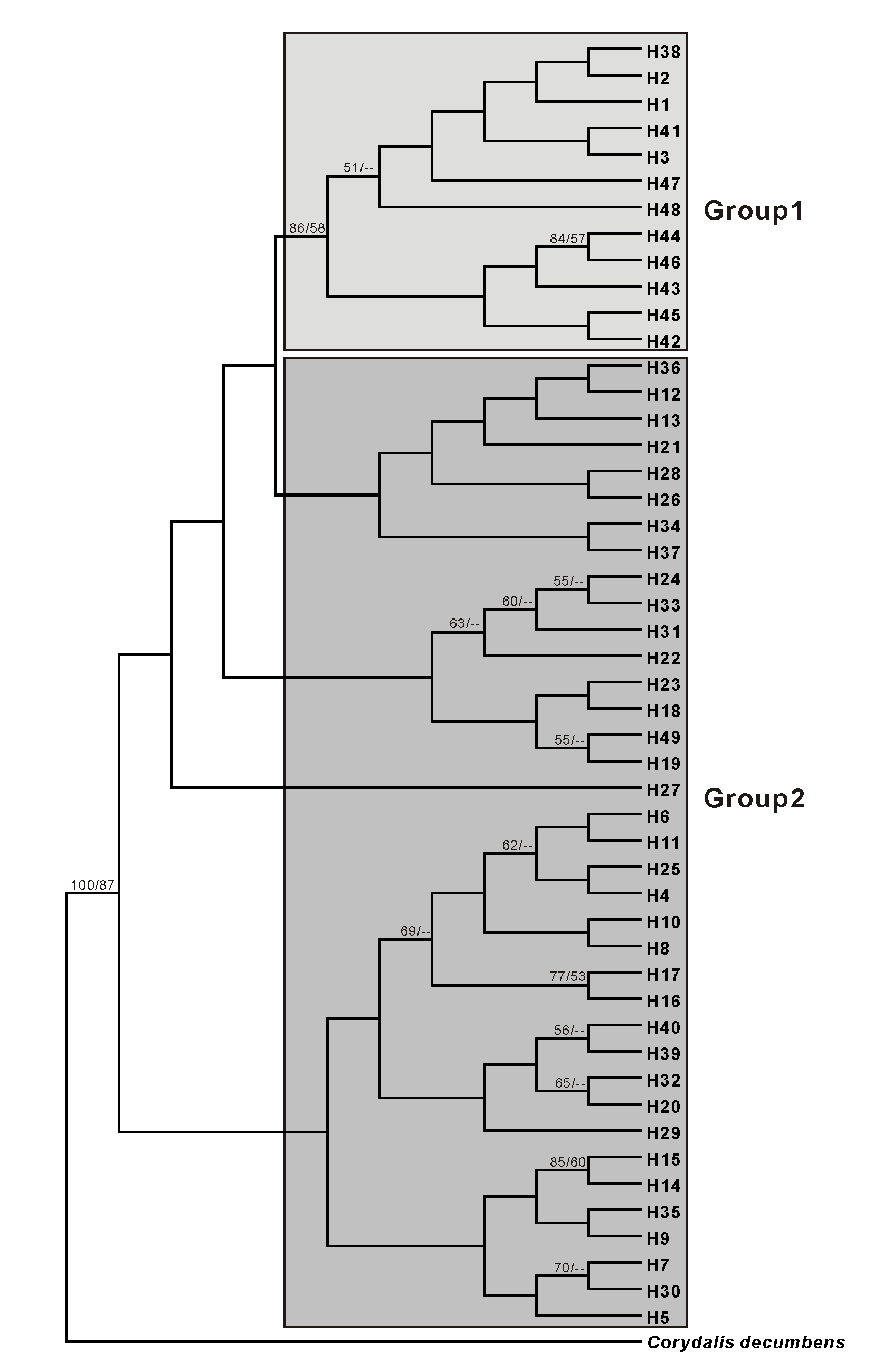


FIGURE S1 | Bayesian consensus tree based on 49 ITS haplotypes identified in *C. hendersonii*. Numbers next to nodes indicated posterior probabilities and bootstrap values (only the values > 50% are shown) based on Bayesian and maximum-likelihood (ML) analysis respectively.
